# Focal Adenomyosis and Uterine Junctional Zone Abnormalities Are Associated With Live Birth Rates in Women With Infertility and Adenomyosis Undergoing Assisted Reproductive Technologies: A Prospective Observational Cohort Study

**DOI:** 10.1155/ogi/7841795

**Published:** 2026-11-13

**Authors:** Shunji Zhang, Yangqin Peng, Yi Tang, Sufen Cai, Xiaofeng Li, Hui Guo, Qin Niu, Xihong Li, Zhengxiang Yi, Jian Wang, Ge Lin, Fei Gong

**Affiliations:** ^1^ Clinical Research Center for Reproduction and Genetics in Hunan Province Changsha China; ^2^ Reproductive & Genetic Hospital of CITIC-Xiangya Changsha China zxxyyy.cn; ^3^ School of Basic Medical Sciences Central South University Changsha China csu.edu.cn; ^4^ Imaging Center Hunan Guangxiu Hospital Changsha China; ^5^ NHC Key Laboratory of Human Stem Cell and Reproductive Engineering Changsha China

**Keywords:** adenomyosis, focal adenomyosis, junctional zone abnormalities, live birth rate, magnetic resonance imaging

## Abstract

**Background and Aims:**

Previous studies have shown that adenomyosis increases the incidence of failure and recurrent miscarriage in patients undergoing in vitro fertilization embryo transfer. This study aimed to evaluate the association between magnetic resonance imaging (MRI) characteristics of women with infertility and adenomyosis and their assisted reproductive technologies (ART) outcomes, as indicated by ongoing pregnancy and live birth rates.

**Methods:**

This prospective observational cohort study included 1679 women diagnosed with adenomyosis via 3D ultrasound who underwent ART between October 2021 and December 2023. Pelvic MRI was performed by two qualified radiologists, and assessments were conducted according to standard diagnostic criteria. Baseline demographics, MRI manifestations, as well as parameters and outcomes of the first embryo transfer cycle were collected. Clinical and imaging factors of live birth in patients were identified. A predictive model for live birth in women with infertility and adenomyosis was developed using multivariable logistic regression and visualized as a nomogram.

**Results:**

In total, 1400 women underwent MRI and completed ART. The mean age of the participants was 31.84 ± 3.56 years. Ongoing pregnancy rate was 58.21% (815/1400), whereas live birth rate was 56.21% (787/1400). Multivariable logistic regression of MRI characteristics identified focal adenomyosis and junctional zone abnormalities as factors associated with live birth rates. A predictive model incorporating six clinical and imaging factors had an area under the receiver operating characteristic curve of 0.640 (95% confidence interval: 0.607–0.673).

**Conclusion:**

This study represents the first large‐scale prospective observational cohort study to explore the relationship between MRI features of adenomyosis and clinical outcomes of ART. Focal adenomyosis and JZ abnormalities were associated with live birth rates in women with adenomyosis undergoing ART; further research is needed to confirm these associations and to explore their clinical utility.

## 1. Background

Adenomyosis is a condition characterized by the benign invasion of endometrial tissue into the myometrium, presenting as either diffuse or localized growth. The mean frequency of adenomyosis at hysterectomy is 20%–30% [1, 2]. The prevalence of adenomyosis in series where the diagnosis was based on imaging is approximately 20%–30% [3, 4], whereas among patients with infertility, the prevalence of adenomyosis ranges from 21% to 33%, rising to nearly 50% in those showing clinical symptoms [5].

Assisted reproductive technologies (ARTs) are essential for managing infertility in patients with adenomyosis. A study has indicated that adenomyosis contributes to 38.2% of in vitro fertilization (IVF) failures and 34.7% of cases of recurrent pregnancy loss [6], underscoring its significant impact on ART outcomes. Additionally, the largest cohort study with a 10‐year time span included 7608 pregnant patients with adenomyosis and 566,153 pregnant women without any history of endometriosis. This study found that adenomyosis was 52.1% more likely to present a complication (OR = 1.521). Specifically, compared to non–endometriosis patients, pregnant women with adenomyosis have a higher incidence of IUGR (OR = 1.257 (95% CI: 1.064–1.485) (*p* = 0.007)); preterm delivery (OR = 1.422 (95% CI: 1.264–1.600) (*p* = 0.0001)); cesarean delivery (OR = 1.099 (95% CI: 1.002–1.205) (*p* = 0.046)); hypertensive disorders (OR = 1.177 (95% CI: 1.076–1.288) (*p* = 0.0001)); abruption placentae (OR = 1.197 (95% CI: 1.008–1.422) (*p* = 0.040)), and spontaneous abortion (OR = 1.529 (95% CI: 1.360–1.718) (*p* = 0.0001)) [7]. Moreover, in 2024, a case‐control study by de Rozario et al. associated adenomyosis with adverse obstetric outcomes, including increased risks of cesarean delivery, intrauterine growth restriction, postpartum hemorrhage, preeclampsia, and prior spontaneous miscarriages [8]. Early and accurate diagnosis of adenomyosis is therefore clinically essential, as it may inform individualized treatment strategies, improve patient counseling, and potentially optimize ART outcomes.

Adenomyosis may exhibit diverse morphological patterns, ranging from mild localized expansion of the junctional zone (JZ) to extensive myometrial hyperplasia and fibrosis, potentially affecting uterine tension and the contour of the endometrial cavity [5]. These variations result in diverse and complex imaging manifestations. While some patients present with subtle and isolated features, others display multiple and severe abnormalities, with various features potentially coexisting or being fully expressed.

Although pathology is considered the “gold standard” for diagnosing adenomyosis, its application is limited by its invasive nature. Moreover, clinical diagnosis for patients with infertility desiring conception relies primarily on symptoms and imaging examinations such as transvaginal ultrasonography (TVUS) and magnetic resonance imaging (MRI). Advanced TVUS techniques, guided by standardized criteria such as the morphological uterus sonographic assessment (MUSA), enable early and accurate diagnosis of adenomyosis, which is crucial for timely patient management and counseling [9]. MRI, a noninvasive and highly accurate diagnostic tool, is often employed as a second‐line examination for diagnosing adenomyosis, with reported sensitivity and specificity of 88%–93% and 67%–91%, respectively [10]. However, the specific impact of individual imaging characteristics on diagnosis and prognosis remains unclear. Additionally, the coexistence of different imaging features may reflect varying degrees of disease severity and exhibit distinct effects on pregnancy outcomes. Consequently, previous studies have explored the relationships between various imaging parameters (e.g., uterine diameters [11], number of imaging abnormalities [12, 13], internal versus external adenomyosis [14, 15], and high‐intensity myometrial spots [16]) and disease diagnosis or prognosis.

Therefore, in this study, we aimed to evaluate the relationship between the MRI characteristics of women with infertility and adenomyosis and their ART outcomes, as indicated by ongoing pregnancy and live birth rates.

## 2. Materials and Methods

### 2.1. Study Design and Participants

This prospective observational cohort study was registered on a clinical research platform and approved by our ethics committee (ethics approval no.: LL‐SC‐2021‐011; approval date: 25 June 2021). Patient recruitment began in October 2021 and concluded in December 2023. All participants have signed written informed consent.

The inclusion criteria were women requiring ART who met the following criteria: aged between 20 and 38 years, body mass index ranging from 18.5 to 28 kg/m^2^, and a diagnosis of adenomyosis via TVUS according to the 2015 and 2018 MUSA criteria [17, 18]. All ultrasound diagnoses were performed by two qualified radiologists. The exclusion criteria were: (1) contraindications for IVF or intracytoplasmic sperm injection in either partner (e.g., poorly controlled type I or type II diabetes; unexplained liver disease or liver dysfunction; renal disease or kidney dysfunction; severe anemia; history of deep vein thrombosis, pulmonary embolism or cerebrovascular accidents; poorly controlled hypertension or diagnosed heart disease; history of cervical, endometrial, or breast cancer; and unexplained vaginal bleeding); (2) any conditions affecting pregnancy outcomes, including reproductive tract anomalies (e.g., septate uterus > 1 cm, bicornuate uterus, unicornuate uterus), untreated hydrosalpinx, uterine fibroids > 4 cm in diameter, endometrial thickness < 8 mm, and pituitary tumors or malignancies in any organ or tissue; (3) uncorrected hyperprolactinemia, thyroid dysfunction, or adrenal disease; and (4) undergoing preimplantation genetic testing cycles.

### 2.2. Study Methods

Eligible patients were informed about the study, who subsequently provided their written consent before participation. Pelvic MRI was performed by two qualified radiologists. Diagnosis of diffuse adenomyosis via MRI was based on three parameters [3, 19]: (1) maximum JZ thickness (JZmax), defined as the maximum thickness of the low‐intensity band at the endometrial–myometrial interface; (2) maximum ratio between JZmax and myometrial thickness (ratio max), defined as the ratio of JZmax to the corresponding myometrial thickness at the same level; and (3) presence of hyperintense regions within the myometrium. Focal adenomyosis manifests on T2WI as oval, irregular, or quasi‐circular masses within the myometrium, with unclear boundaries and low signal intensity similar to the JZ. Scattered punctate or patchy high signal intensity can also be seen within the lesion, manifesting as cystic dilation or bleeding within the lesion [19]. Additionally, basic demographics and MRI features were recorded, including JZmax, presence of hyperintense regions within the myometrium, focal adenomyosis (total number, mean diameter of the largest adenomyosis, and number of adenomyosis > 3 cm), and JZ abnormalities (e.g., indistinct or poorly defined JZ and finger‐like projections).

Ovarian stimulation protocols including antagonist, long, and ultralong regimens were also recorded. Follicle‐stimulating hormone doses were adjusted based on ovarian function, ranging between 112.5 and 300 IU. Triggering was initiated when follicles with a diameter ≥ 18 mm accounted for more than 50% of all follicles with a diameter ≥ 12 mm. Embryos and blastocysts were scored according to the Gander grading system [20]. Specifically, high‐quality embryos were defined as embryos ≥ 6‐cell, Grade II, or blastocysts ≥ 4 BB. A maximum of two embryos were transferred per cycle. Frozen embryo transfer protocols included natural, hormone replacement, or gonadotropin‐releasing hormone agonist (GnRHa) pretreatment cycles.

Comprehensive data were collected post‐assisted reproduction for a single completed transfer cycle (fresh or frozen), including baseline endocrine profile, antral follicle count, ovarian stimulation protocol and duration, total dose of stimulation agent, hormone levels and follicle count on trigger day, oocyte yield, number of mature oocytes, fertilized oocytes, high‐quality embryos, and high‐quality blastocysts, blastocyst culture outcomes, fresh or frozen transfer details, embryo grading, and endometrial thickness at transfer for both fresh and frozen transfers. Additionally, pregnancy‐related outcomes were tracked up to live birth for pregnant participants, whereas nonpregnant patients left the study following one transfer cycle.

The primary and secondary outcomes were the live birth and ongoing pregnancy rates, respectively. Ongoing pregnancy was defined as the detection of a viable intrauterine fetus on ultrasound 68–70 days post‐transfer. Live birth was defined as the delivery of a live infant at ≥ 28 weeks of gestation.

### 2.3. Statistical Analysis

Data analysis and visualization were conducted using the *R* software (version 3.6.3). LASSO regression was implemented using the glmnet package. Nomogram construction, calibration curve generation, and calculation of the concordance index were carried out via the rms package. Receiver‐operating characteristic curve analysis was performed with the pROC package. Decision curve analysis was conducted using the rmda package. Continuous variables were analyzed using Wilcoxon rank sum tests; noncontinuous variables were assessed using Fisher’s exact tests and Pearson’s Chi‐squared tests. All tests were two‐sided with a priori significance level set at *p* < 0.05. Clinical and imaging predictors of live birth in patients with adenomyosis were identified using least absolute shrinkage and selection operator regression. Based on these results, multivariate stepwise logistic regression was performed to construct a predictive model to predict live births in women with infertility and adenomyosis, which was visualized as a nomogram. The discriminatory ability of the model was evaluated by plotting the receiver operating characteristic curve and calculating the area under the curve, while its calibration was assessed using a calibration curve (generated through bootstrap resampling with 1000 iterations). Additionally, the concordance index was computed to quantify the discrimination and goodness of fit of the model. The clinical utility of the model was determined using decision curve analysis. Given the exploratory nature of the regression analyses, no multiple comparison corrections were applied.

## 3. Results

In this study, 1679 women diagnosed with adenomyosis via 3D ultrasound and undergoing ART met the inclusion criteria, of whom 1400 diagnosed with adenomyosis via MRI and were included in the analysis. The baseline characteristics of enrolled patients are presented in Table 1. The MRI features of adenomyosis are detailed in Table 2. ART parameters and pregnancy outcomes are summarized in Table 3. The mean age of the participants was 31.84 ± 3.56 years. Ongoing pregnancies were recorded in 815 women (ongoing pregnancy rate: 58.21%), and 787 women had live births, indicating a live birth rate of 56.21%.

**TABLE 1:** Baseline characteristics of patients with adenomyosis.

Variable	Overall, *N* = 1,400
Age	31.84 ± 3.56
BMI (kg/m^2^)	22.11 ± 2.10
AMH (ng/mL)	4.35 ± 5.28
Gravidity	1.32 ± 1.42
Parity	1.43 ± 1.46
Repeated pregnancy loss	73 (5.2%)
Repeated implantation failures	17 (1.2%)
Moderate to severe intrauterine adhesions	208 (15%)
Untreated hydrosalpinx	100 (7.1%)
Chronic endometritis	4 (0.3%)
Scarred uterus	175 (13%)
Metabolic disease	734 (52%)
Uterine malformation	12 (0.9%)
Male factors	1399 (100%)

*Note:* Moderate intrauterine adhesions: A score of 5–8 according to the American Reproductive Society (AFS) scoring criteria. Severe intrauterine adhesions: A score of 9–12 according to the American Reproductive Society (AFS) scoring criteria.

**TABLE 2:** The MRI features of patients with adenomyosis.

Variable	Overall, *N* = 1,400
Anteroposterior diameter of uterus	47.28 ± 10.09
Transverse diameter of uterus	53.82 ± 9.12
Longitudinal diameter of uterus	53.44 ± 11.79
Average diameter of uterus	50.36 ± 8.36
Thickness of anterior wall	16.77 ± 4.41
Thickness of JZ in the anterior uterine wall	9.19 ± 6.29
Thickness of posterior wall	20.34 ± 6.26
Thickness of JZ in the posterior uterine wall	10.96 ± 5.98
Thickness of fundal wall	14.40 ± 4.30
Maximum JZ thickness	12.86 ± 6.42
Focal adenomyosis	305 (27%)
Diffuse adenomyosis	104 (9.1%)
Focal combined with diffuse adenomyosis	187 (16%)
Abnormal JZ	320 (23%)
High intensity spots in the myometrium	260 (27%)
Cysts in the myometrium	159 (16%)

**TABLE 3:** ART parameters and pregnancy outcomes of patients with adenomyosis.

Variable	Overall, *N* = 1,400
Number of follicles	11.78 ± 4.02
Number of retrieved oocytes	12.18 ± 5.28
Number of embryos transferred	1.49 ± 0.50
Number of 2PN	7.70 ± 3.83
Number of high‐quality embryos	4.57 ± 3.40
Number of blastocysts	4.00 ± 3.45
Number of high‐quality blastocysts	1.10 ± 1.69
Number of high‐quality embryos transferred	
0	431 (31%)
1	566 (40%)
2	403 (29%)
Endometrial thickness (mm)	11.88 ± 2.13
Frozen embryo transfer	727 (52%)
Fresh embryo transfer	673 (48%)
Ongoing pregnancy	815 (58.21%)
Live birth	787 (56.21%)

Univariate logistic regression analyses were performed to compare clinical and MRI parameters between women who achieved live births and those who did not. The results revealed significant differences in age, number of follicles, anti‐Müllerian hormone levels, gravidity, repeated pregnancy loss, number of embryos transferred, two pronucleus zygote (2PN), high‐quality blastocysts, and transferred high‐quality embryos, endometrial thickness, focal adenomyosis, focal combined with diffuse adenomyosis, abnormal JZ, presence of moderate‐to‐severe intrauterine adhesions and a scarred uterus, and fresh embryo transfer. Conversely, no significant differences were observed in the uterine diameters (anteroposterior, transverse, longitudinal, and average), uterine wall thickness (anterior, posterior, and fundal), and JZ measurements (anterior wall, posterior wall, and maximum thicknesses). Detailed results are provided in Tables 4 and 5.

**TABLE 4:** Comparison of clinical and MRI parameters between women who achieved live births and those who did not.

Category	Variable	Live birth (−), *N* = 613^1^	Live birth (+), *N* = 787^1^	*p*‐value^2^
Maternal characteristics	Age	32.28 ± 3.49	31.50 ± 3.58	< 0.001
	BMI (kg/m^2^)	22.00 ± 2.04	22.20 ± 2.15	0.073
	AMH (ng/mL)	3.95 ± 3.16	4.66 ± 6.45	< 0.001
	Gravidity	1.58 ± 1.54	1.32 ± 1.38	0.002
	Parity	0.33 ± 0.57	0.29 ± 0.56	0.082
	Repeated pregnancy loss	43 (7.0%)	30 (3.8%)	0.007
	Repeated implantation failures	9 (1.5%)	8 (1.0%)	0.444
	Moderate to severe intrauterine adhesions	111 (18%)	97 (12%)	0.003
	Untreated hydrosalpinx	40 (6.5%)	60 (7.6%)	0.428
	Chronic endometritis	3 (0.5%)	1 (0.1%)	0.325
	Scarred uterus	98 (16%)	77 (9.8%)	< 0.001
	Metabolic disease	309 (50%)	425 (54%)	0.181
	Uterine malformation	5 (0.8%)	7 (0.9%)	0.882
	Male factors	612 (100%)	787 (100%)	0.438

MRI features	Anteroposterior diameter of uterus	47.40 ± 9.44	47.18 ± 10.61	0.250
	Transverse diameter of uterus	53.37 ± 9.00	54.20 ± 9.21	0.080
	Longitudinal diameter of uterus	53.03 ± 11.93	53.78 ± 11.66	0.342
	Average diameter of uterus	50.22 ± 8.25	50.49 ± 8.46	0.649
	Thickness of anterior wall	16.82 ± 4.45	16.72 ± 4.38	0.600
	Thickness of JZ in the anterior uterine wall	8.89 ± 4.61	9.45 ± 7.41	0.482
	Thickness of posterior wall	20.31 ± 6.21	20.37 ± 6.30	0.940
	Thickness of JZ in the posterior uterine wall	11.14 ± 6.04	10.82 ± 5.94	0.465
	Thickness of fundal wall	14.29 ± 4.34	14.50 ± 4.27	0.348
	Maximum JZ thickness	12.69 ± 4.79	13.01 ± 7.55	0.469
	Focal adenomyosis	143 (31%)	162 (24%)	0.005
	Diffuse adenomyosis	50 (11%)	54 (7.9%)	0.086
	Focal combined with diffuse adenomyosis	89 (19%)	98 (14%)	0.024
	Abnormal JZ	158 (26%)	162 (21%)	0.022
	High‐intensity spots in the myometrium	122 (27%)	138 (26%)	0.603
	Cyst in myometrium	74 (17%)	85 (16%)	0.803

Reproductive parameters	Number of follicles	11.44 ± 4.22	12.05 ± 3.84	0.002
	Number of retrieved oocytes	11.88 ± 5.22	12.42 ± 5.32	0.087
	Number of embryos transferred	1.44 ± 0.50	1.53 ± 0.50	< 0.001
	Number of 2PN	7.37 ± 3.80	7.96 ± 3.84	0.004
	Number of high‐quality embryos	4.38 ± 3.45	4.72 ± 3.36	0.011
	Number of blastocysts	3.81 ± 3.29	4.15 ± 3.56	0.120
	Number of high‐quality blastocysts	0.99 ± 1.53	1.18 ± 1.81	0.366
	Number of high‐quality embryos transferred			0.052
	0	198 (32%)	233 (30%)	
	1	259 (42%)	307 (39%)	
	2	156 (25%)	247 (31%)	
	Endometrial thickness (mm)	11.70 ± 2.17	12.03 ± 2.09	0.004
	Frozen or fresh embryo transfer			< 0.001
	Frozen embryo transfer	357 (58%)	370 (47%)	
	Fresh embryo transfer	256 (42%)	417 (53%)	

*Note:* Moderate intrauterine adhesions: A score of 5–8 according to the American Reproductive Society (AFS) scoring criteria. Severe intrauterine adhesions: A score of 9–12 according to the American Reproductive Society (AFS) scoring criteria.

^1^Mean ± SD or frequency (%).

^2^Wilcoxon rank sum test; Fisher’s exact test; Pearson’s Chi‐squared test.

**TABLE 5:** Univariate logistic regression of clinical and MRI parameters between women who achieved live births and those who did not.

Category	Characteristic	OR	95% CI	*p*‐value
Maternal characteristics	Age	0.94	0.91, 0.97	< 0.001
	BMI (kg/m^2^)	1.05	1.00, 1.10	0.076
	AMH (ng/mL)	1.06	1.02, 1.10	0.003
	Gravidity	0.89	0.82, 0.95	0.001
	Parity	0.88	0.73, 1.06	0.17
	Repeated pregnancy loss	0.53	0.32, 0.84	0.008
	Repeated implantation failures	0.69	0.26, 1.81	0.45
	Moderate to severe intrauterine adhesions	0.64	0.47, 0.85	0.003
	Untreated hydrosalpinx	1.18	0.78, 1.80	0.43
	Chronic endometritis	0.26	0.01, 2.03	0.24
	Scarred uterus	0.57	0.41, 0.78	< 0.001
	Metabolic disease	1.16	0.93, 1.43	0.18
	Uterine malformation	1.09	0.35, 3.70	0.88
	Male factors	0.90	0.72, 1.12	0.34

MRI features	Anteroposterior diameter of uterus	1.00	0.99, 1.01	0.73
	Transverse diameter of uterus	1.01	1.00, 1.02	0.16
	Longitudinal diameter of uterus	1.01	1.0, 1.02	0.33
	Average diameter of uterus	1.00	0.99, 1.02	0.62
	Thickness of anterior wall	1.00	0.97, 1.03	0.75
	Thickness of JZ in the anterior uterine wall	1.02	0.99, 1.05	0.26
	Thickness of posterior wall	1.00	0.98, 1.02	0.87
	Thickness of JZ in the posterior uterine wall	0.99	0.97, 1.02	0.49
	Thickness of fundal wall	1.01	0.98, 1.04	0.46
	Maximum JZ thickness	1.01	0.99, 1.03	0.51
	Focal adenomyosis	0.69	0.53, 0.90	0.006
	Diffuse adenomyosis	0.70	0.47, 1.05	0.087
	Focal adenomyosis combined with diffuse adenomyosis	0.70	0.51, 0.96	0.025
	Abnormal JZ	0.75	0.58, 0.96	0.022
	High‐intensity spots in the myometrium	0.93	0.70, 1.23	0.60
	Cyst in myometrium	0.96	0.68, 1.35	0.80

Reproductive parameters	Number of follicles	1.04	1.01, 1.07	0.005
	Number of retrieved oocytes	1.02	1.00, 1.04	0.060
	Number of embryos transferred	1.44	1.16, 1.78	< 0.001
	Number of 2PN	1.04	1.01, 1.07	0.005
	Number of high‐quality embryos	1.03	1.00, 1.06	0.064
	Number of blastocysts	1.03	1.00, 1.06	0.067
	Number of high‐quality blastocysts	1.07	1.00, 1.14	0.041
	Number of high‐quality embryos transferred	1.16	1.01, 1.33	0.038
	Endometrial thickness (mm)	1.08	1.03, 1.14	< 0.001
	Frozen or fresh embryo transfer			
	Frozen embryo transfer	—	—	
	Fresh embryo transfer	1.57	1.27, 1.95	< 0.001

*Note:* Moderate intrauterine adhesions: A score of 5–8 according to the American Reproductive Society (AFS) scoring criteria. Severe intrauterine adhesions: A score of 9–12 according to the American Reproductive Society (AFS) scoring criteria.

Abbreviations: CI = confidence interval, OR = odds ratio.

Subsequently, multivariate logistic regression analyses were performed to compare clinical and MRI parameters between women who achieved live births and those who did not. The results identified focal adenomyosis and JZ abnormalities as factors associated with live births in patients with adenomyosis (Table 6). MRI images of focal adenomyosis, and abnormal JZ are shown in Supporting Figures 1 and 2. Additionally, multivariate stepwise logistic regression identified six clinical and imaging factors of live birth rate in patients with adenomyosis. These factors were used to construct a predictive model to predict live births in women with infertility and adenomyosis, which was visualized as a nomogram, as shown in Figure 1. The six factors included in the model were the number of transferred embryos, focal adenomyosis, JZ abnormalities, number of good blastocysts, presence of a scarred uterus, repeated pregnancy loss, and cycle type (fresh vs. frozen embryo transfer). The area under the curve of the model was 0.640 (95% CI: 0.607–0.673) (Figure 2). As presented in Figure 3, the calibration curve indicated agreement between model‐predicted and observed live‐birth rates for women with adenomyosis. Additionally, the clinical utility of the model was confirmed by decision curve analysis (Figure 4).

**TABLE 6:** Multivariate logistic regression of clinical and MRI parameters between women who achieved live births and those who did not.

Characteristic	OR	95% CI	*p*‐value
Age	0.97	0.93, 1.01	0.13
AMH (ng/mL)	1.01	0.97, 1.06	0.70
Gravidity	1.01	0.91, 1.12	0.84
Repeated pregnancy loss	0.52	0.28, 0.96	0.038
Moderate to severe intrauterine adhesions	0.76	0.51, 1.13	0.18
Scarred uterus	0.75	0.50, 1.14	0.18
Focal adenomyosis	0.65	0.48, 0.87	0.004
Focal combined with diffuse adenomyosis	0.72	0.50, 1.03	0.067
Abnormal JZ	0.69	0.51, 0.92	0.010
Number of follicles	1.01	0.96, 1.05	0.77
Number of 2PN	1.00	0.95, 1.05	0.95
Number of high‐quality blastocysts	1.16	1.06, 1.28	0.002
Number of embryos transferred	1.70	1.26, 2.31	< 0.001
Number of high‐quality embryos transferred	0.94	0.78, 1.13	0.50
Endometrial thickness (mm)	1.03	0.98, 1.09	0.24
Frozen or fresh embryo transfer			
Frozen embryo transfer	—	—	
Fresh embryo transfer	1.66	1.24, 2.24	< 0.001

*Note:* Perform multivariate logistic regression analysis on indicators with *p* < 0.05 included in univariate analysis. Moderate intrauterine adhesions: A score of 5–8 according to the American Reproductive Society (AFS) scoring criteria. Severe intrauterine adhesions: A score of 9–12 according to the American Reproductive Society (AFS) scoring criteria.

Abbreviations: CI = confidence interval, OR = odds ratio.

**FIGURE 1:**
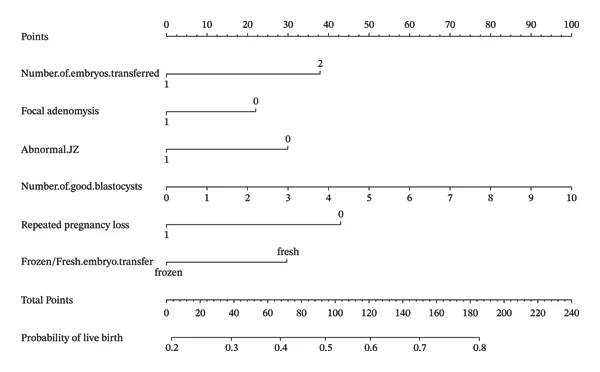
Nomogram model for predicting live births of women with adenomyosis. To predict the probability of live births, add the points of the six predictors together to get the “total point”. A straight line is drawn from the “total point” axis down to the “probability of live births” axis to determine the risk of live birth of women with adenomyosis.

**FIGURE 2:**
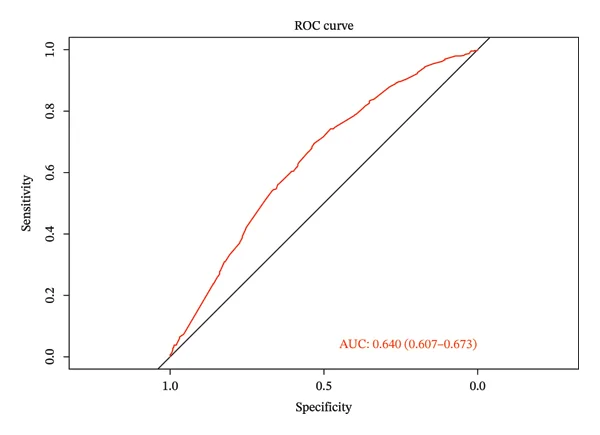
The ROC curve of the risk of live births predicted by the nomogram model. The area under the curve of the model was 0.640 (95% CI: 0.607–0.673).

**FIGURE 3:**
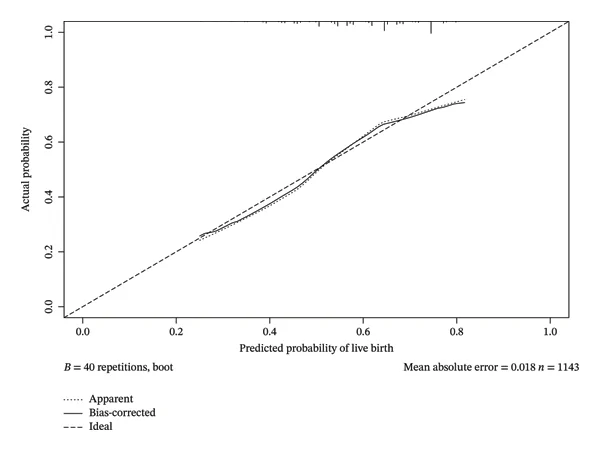
The calibration curve of the nomogram for predicting the risk of live births of women with adenomyosis.

**FIGURE 4:**
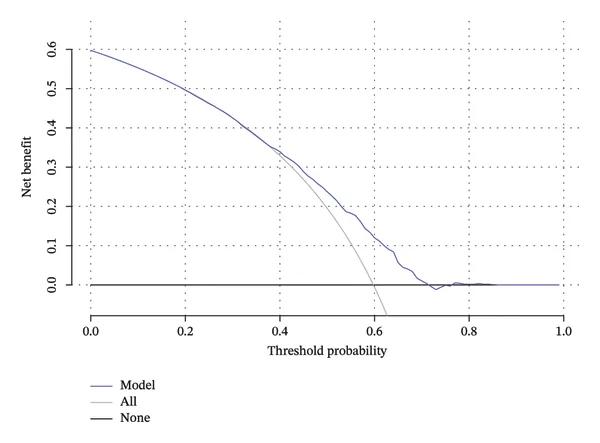
The decision curve of the nomogram for predicting the risk of live births of women with adenomyosis.

## 4. Discussion

To the best of our knowledge, this study represents the first large‐scale prospective observational cohort study to explore the relationship between MRI‐detected features of adenomyosis and clinical outcomes of ART, indicated by live birth rates. We found that focal adenomyosis and JZ abnormalities are associated with live birth rates in adenomyosis. Additionally, our predictive model, developed by incorporating clinical and imaging factors, had an AUC of 0.640.

Most existing studies suggest that women with adenomyosis experience higher miscarriage rates and lower ongoing pregnancy and birth rates [6, 21]. In a meta‐analysis conducted in 2017, Younes et al. reviewed a total of 18 studies, including nine clinical studies, four prospective cohort studies, and five retrospective cohort studies. Among these studies, six reported that the IVF miscarriage rate was higher in women with adenomyosis, while three studies reported no significant impacts. This discrepancy may stem from the lack of classification or staging of adenomyosis in these studies, as well as differences in the characteristics of the study cohort and the unknown severity of the condition, making it challenging to draw objective and accurate conclusions [6]. Nonetheless, in 2018, Stanekova et al. reported that, after excluding embryo factors, the miscarriage rate in women with adenomyosis was significantly higher than that in those without [21].

Thus, researchers have long been focused on identifying factors and indicators that influence pregnancy outcomes. Accordingly, some studies suggest that JZ thickening in women with adenomyosis negatively impacts pregnancy outcomes. Three distinct layers can be distinguished in the T2‐weighted images of women of reproductive age: the endometrium with a high intensity, the outer myometrium with an intermediate intensity, and a low‐intensity band in between, referred to as the JZ. The JZ, a unique myometrial band‐like structure, was first described by Hricak et al. in 1983 on MRI [22]. A comparison of histological measurements of excised uterine tissue with MRI data revealed that the hyperintense layer on MRI corresponded to the endometrial thickness. Conversely, the low‐intensity band only contained the innermost myometrium, characterized by increased cellular density, corresponding to the thickness of the JZ. Subsequently, a more recent study further confirmed the match between the MRI‐identified JZ and the histological inner myometrium [23]. Additionally, a retrospective study using the MUSA criteria to diagnose the outcome of donor‐assisted pregnancy in patients with adenomyosis showed that uterine volume and diffuse uterine muscle thickening are not related to assisted reproductive outcomes. Instead, the increase in focal lesion diameter is significantly correlated with a decrease in live birth rate, with a decrease coefficient of 0.91; for every 1 cm increase in lesion diameter, the birth rate decreases by 11 times [24]. In contrast to the above results, a prospective observational study on the impact of adenomyosis on IVF outcomes in women undergoing donor oocyte transfer in 2024 analyzed the effects of focal adenomyosis, diffuse adenomyosis, or adenomyoma on assisted reproductive outcomes. The results showed that patients with JZ abnormalities had a relative risk (RR) of miscarriage that increased by more than three times (RR, 3.28; 95% confidence interval [CI], 1.38‐7.78). Moreover, the risk of miscarriage doubled in patients with diffuse JZ thickening and severe adenomyosis, while no significant difference was observed in patients with focal adenomyosis [25].

Furthermore, in 2010, Piver et al. explored the relationship between MRI‐detected JZ thickness and IVF outcomes, and found a negative correlation between JZ thickness and IVF implantation rates. On this basis, they suggested that MRI‐detected JZ thickness could serve as the optimal negative predictor for implantation failure [26]. Thereafter, in recent years, multiple studies have indicated that JZ thickening is associated with implantation failure and mitigated pregnancy rates. After controlling for factors such as advanced age, uterine anomalies, and thin endometrial thickness, studies showed that when the number of transferred embryos, number of high‐quality embryos, and pre‐transfer endometrial thickness were consistent, the clinical pregnancy rate was significantly lower in the group with a JZmax ≥ 12 mm compared to those with a JZmax < 12 mm [27, 28]. Conversely, the results of this study did not identify JZ thickness as an independent factor influencing the ongoing pregnancy rate or birth rate in women with adenomyosis undergoing assisted reproduction.

Our multiple regression analysis identified that focal adenomyosis was independently associated with live birth rates among infertile patients undergoing ART. However, unlike previous literature, our study did not find a relationship between the thickness of the JZ and adverse live birth outcomes. Instead, the results of this study revealed that JZ abnormalities on MRI, such as indistinct or difficult‐to‐observe areas and finger‐like indentations, were independent factors influencing live birth outcomes in women with adenomyosis undergoing assisted reproduction.

Most studies and clinicians rely on three main MRI criteria for diagnosing adenomyosis, namely, JZ thickness, JZ‐to‐myometrium ratio, and the presence of hyperintense regions in the myometrium. However, in 2017, Lisa Agostinho et al. [19] reported several MRI manifestations of adenomyosis beyond these three primary criteria. Additionally, JZ interruption and irregular morphology on 3D ultrasound are listed as ultrasound diagnostic criteria for adenomyosis. Moreover, in 2023, Harmsen et al. [29] described a comparison of MRI and ultrasound features in women with adenomyosis, noting that high‐signal intensity spots in the JZ or myometrium on T2‐weighted images indicated ectopic endometrial tissue, cystic dilation of endometrial glands, or hemorrhagic lesions. Meanwhile, on transvaginal ultrasound, this appeared as sub‐endometrial high‐echo lines and buds, JZ interruptions, or hypoechoic cystic areas in the central or external myometrium. Therefore, other JZ abnormalities should be considered for inclusion in the MRI diagnostic criteria in addition to JZ thickening. Thus, our findings provide supporting evidence that JZ morphological abnormalities beyond JZ thickening merit further consideration when interpreting MRI features of adenomyosis.

In 2021, an observational cohort study on the MRI manifestations of women with adenomyosis revealed that 109 participants (44.0%) presented exclusively with external adenomyosis. Specifically, the lesion was located in the outer myometrium, separated from the endometrium by an intact JZ and myometrium. Conversely, 78 participants (31.5%) had internal adenomyosis, marked by JZ abnormalities, while maintaining a normal outer myometrium [13]. Owing to the unique anatomical structure of the endometrium, which lacks a submucosal layer for tissue protection, the basal layer and glands are in direct contact with the myometrium. Therefore, repeated endometrial injuries may compromise the integrity of the JZ. Moreover, stromal trophoblasts invade the decidua during embryo implantation. This process triggers initial JZ remodeling, extracellular matrix alterations, uterine smooth muscle relaxation, and spiral artery remodeling, thereby facilitating deep trophoblast invasion [30]. Thus, deficiencies or incomplete remodeling of the inner myometrium are among the contributors to implantation failure. Therefore, in addition to the inflammatory environment caused by adenomyosis, structural changes in the JZ may impair decidualization, leading to inadequate spiral artery remodeling. This disruption in uterine physiology may compromise embryo implantation and successful pregnancy [31].

Moreover, previous research has highlighted a negative correlation between uterine volume and infertility outcomes. A 2023 retrospective cohort study on patients with adenomyosis diagnosed via TVUS undergoing IVF treatment [11] reported that an increased uterine volume was associated with higher miscarriage rates and lower live birth rates. The study identified a uterine volume at 8 weeks of gestation (90–130 cm^3^ for 6–8 weeks of gestation) as a turning point for higher miscarriage rates, as well as a uterine volume at 10 weeks of gestation (130–180 cm^3^ for 8–10 weeks of gestation, or > 180 cm^3^ for more than 10 weeks of gestation) as a turning point for lower live birth rates. Additionally, their analysis indicated that patients with a uterus larger than at 8 weeks of gestation exhibited significantly higher miscarriage rates, along with lower live birth and cumulative live birth rates across all embryo transfer cycles (fresh and frozen). In contrast, the results of this study did not identify uterine volume as a significant factor influencing pregnancy or live birth outcomes in patients with adenomyosis undergoing assisted reproduction. However, this does not entirely rule out the potential impact of uterine dimensions or JZ thickening on reproductive outcomes. This is because it is possible that patients with larger uterine dimensions in this study may have received more clinical attention, thereby having received appropriate GnRHa pretreatment based on prior studies and clinical experience. Conversely, those with isolated JZ abnormalities identified on MRI, who were perceived as having “mild” conditions, might have been overlooked for GnRHa therapy. Consequently, these findings underscore the need for further randomized controlled trials to explore whether GnRHa pretreatment is beneficial for patients with isolated JZ abnormalities and to determine the optimal pretreatment duration for improving reproductive outcomes.

The strengths of this study include its large‐scale and prospective study design. Additionally, our results revealed that focal adenomyosis and abnormal JZs were associated with the live birth rate of women with adenomyosis. Based on our findings, clinicians may consider heightened awareness of JZ abnormalities and focal adenomyosis lesions among infertile women undergoing ART. Women with these features might benefit from closer monitoring and individualized treatment planning; however, the potential value of GnRHa pretreatment in this subgroup remains to be confirmed in future randomized controlled trials. Notably, previous studies have consistently reported reduced live birth rates in women with adenomyosis. Furthermore, Brosens et al. highlighted that implantation and placental abnormalities were key mechanisms underlying severe pregnancy complications, including late miscarriage, preterm birth, fetal growth restriction, and preeclampsia. These issues are attributed to deep placental damage caused by defective spiral artery remodeling in the JZ [32]. Further randomized controlled trials are warranted to develop targeted interventions for improving pregnancy outcomes in patients with focal adenomyosis and JZ abnormalities.

It is noteworthy that adenomyosis frequently coexists with endometriosis, and both conditions independently and synergistically contribute to adverse fertility and obstetric outcomes. This overlap underscores the importance of comprehensive pelvic evaluation in infertile women, as combined disease may necessitate tailored multidisciplinary management [33].

## 5. Reflections

This study had some limitations. First, the participants were selected after a TVS scan and MRI showing signs of adenomyosis, although ultrasound and MRI have good specificity and sensitivity in the diagnosis of adenomyosis. On the other hand, the AUC of the model was rather modest, despite calibration: six indicators showed significant differences in the multivariate analysis, and only these six indicators were included in the multivariate model. Our multiple regression analysis showed no statistical difference in age, and thus, it was not included in the model analysis. However, the reason for this may be that the included patients were aged 20–38 years, which is relatively young among patients with infertility. It should also be acknowledged that potential confounders such as differences in ART protocols, use of hormonal pretreatments, and incomplete data on prior surgical history could not be comprehensively adjusted for in this observational study, and these factors may have influenced the observed associations. Third, we excluded patients with endometrial thickness of < 8 mm, because we were mainly concerned about the thin endometrium caused by moderate‐to‐severe intrauterine adhesions, as well as other uterine factors that significantly affect implantation. However, this might have excluded a subset of patients where the adenomyosis affects endometrial proliferation. Fourth, the observational design precludes causal inferences, and the heterogeneous patient population may affect generalizability. Further research should include propensity matching to analyze differences in pregnancy outcomes between patients with adenomyosis and those without adenomyosis. Fifth, while our study identifies key MRI features associated with live birth, we were unable to perform subgroup analyses based on the coexistence or phenotype of endometriosis, as this information was not systematically available for a significant portion of our cohort. Given the known interplay between these two conditions, the absence of this stratification represents a limitation, and future studies with comprehensive surgical and histological phenotyping are warranted to delineate their combined effects on ART outcomes. Additionally, the lack of multiple comparison corrections in our regression models may increase the risk of Type I error, which should be considered when interpreting the findings.

## Author Contributions

Shunji Zhang, Yangqin Peng, and Fei Gong conceived and coordinated the study; designed, performed, and analyzed the experiments; and wrote the paper. Yi Tang, Sufen Cai, Xiaofeng Li, Hui Guo, Qin Niu, Xihong Li, Zhengxiang Yi, Jian Wang, and Ge Lin conducted the data collection, data analysis, and revised the paper.

## Funding

This work was supported by the Hunan Provincial Grant for Innovative Province Construction (grant number 2019SK4012) and the research grant of CITIC‐Xiangya (grant number YWXM‐202205).

## Disclosure

All authors reviewed the results and approved the final version of the manuscript. Professor Gong Fei had full access to all of the data in this study and takes complete responsibility for the integrity of the data and the accuracy of the data analysis. The funders had no role in study design, data collection, analysis, interpretation, writing of the report, or the decision to submit the report for publication.

## Ethics Statement

The study protocol was approved by the Reproductive and Genetic Hospital of CITIC‐XIANGYA’s ethics committee to ensure that all research activities were conducted in accordance with the ethical standards (approval date: June 25 2021).

## Conflicts of Interest

The authors declare no conflicts of interest.

## Supporting Information

Additional supporting information can be found online in the Supporting Information section.

## Supporting information


**Supporting Information 1** Supporting Figure 1. Focal adenomyosis. Sagittal T2‐weighted image; focal asymmetric thickening of the junctional zone forming an ill‐defined area of low signal intensity.

**Supporting Information 2** Supporting Figure 2. Abnormal JZ. Sagittal T2‐weighted images; thickened junctional zone with striated high‐signal intensity areas radiating from the endometrium toward the myometrium (yellow arrow).

## Data Availability

The data that support the findings of this study are available from the corresponding author upon reasonable request, subject to institutional ethics approval, as the data contain potentially identifying patient information.
